# Optimizing Imaging Conditions for Demanding Multi-Color Super Resolution Localization Microscopy

**DOI:** 10.1371/journal.pone.0158884

**Published:** 2016-07-08

**Authors:** Leila Nahidiazar, Alexandra V. Agronskaia, Jorrit Broertjes, Bram van den Broek, Kees Jalink

**Affiliations:** 1 Cell Biophysics group, Department of Cell biology, The Netherlands Cancer Institute, Amsterdam, The Netherlands; 2 Molecular Biophysics group, Utrecht University, Utrecht, the Netherlands; 3 Faculty of Science, University of Amsterdam, Amsterdam, The Netherlands; 4 Van Leeuwenhoek Centre for Advanced Microscopy, Molecular Cytology, Swammerdam Institute for Life Sciences, University of Amsterdam, Amsterdam, The Netherlands; University of Pécs Medical School, HUNGARY

## Abstract

Single Molecule Localization super-resolution Microscopy (SMLM) has become a powerful tool to study cellular architecture at the nanometer scale. In SMLM, single fluorophore labels are made to repeatedly switch on and off (“blink”), and their exact locations are determined by mathematically finding the centers of individual blinks. The image quality obtainable by SMLM critically depends on efficacy of blinking (brightness, fraction of molecules in the on-state) and on preparation longevity and labeling density. Recent work has identified several combinations of bright dyes and imaging buffers that work well together. Unfortunately, different dyes blink optimally in different imaging buffers, and acquisition of good quality 2- and 3-color images has therefore remained challenging. In this study we describe a new imaging buffer, OxEA, that supports 3-color imaging of the popular Alexa dyes. We also describe incremental improvements in preparation technique that significantly decrease lateral- and axial drift, as well as increase preparation longevity. We show that these improvements allow us to collect very large series of images from the same cell, enabling image stitching, extended 3D imaging as well as multi-color recording.

## Introduction

When viewed by conventional fluorescence light microscopy even the smallest details in a cell will be visualized as little blurry 'blobs' of light due to light diffraction. Consequently, closely spaced cellular details cannot be resolved individually because the fluorescence blobs (approximately 250 nm in X and Y, and approximately 650 nm in Z) overlap in the image. Localization-based super resolution microscopy methods (STORM, GSDIM, PALM and variants thereof) circumvent this diffraction barrier by ensuring that at any moment in time the majority of fluorophores are in a dark *off-state* while only a few clearly separable fluorophores are in the bright *on-state*. By finding the center of all these fluorescent blobs (which are termed 'events' or 'blinks') in many thousands of sequential images, the molecules can be localized much more precisely, resulting in up to 10-fold improved resolution. Localization microscopy is therefore rapidly gaining popularity for the study of the ultrastructure of cells. It can be implemented with relatively simple instrumentation: a good fluorescence microscope with excellent stability (i.e., extremely low mechanical drift over time), a sensitive EM-CCD or CMOS digital camera and powerful lasers for excitation are the main ingredients.

In GSDIM and dSTORM microscopy, cells are typically labeled densely with synthetic fluorescent labels, either through conjugation to (secondary) antibodies for immunolabeling, or to specific marker molecules such as phalloidin, which labels actin filaments. Imaging starts with all molecules in the on-state. Using very intense excitation (laser power typically 50–150 mW at the preparation) the majority of fluorophores is rapidly pushed in the dark state, allowing for the precise localization of the few remaining molecules by software fitting.

**Fig 1:**
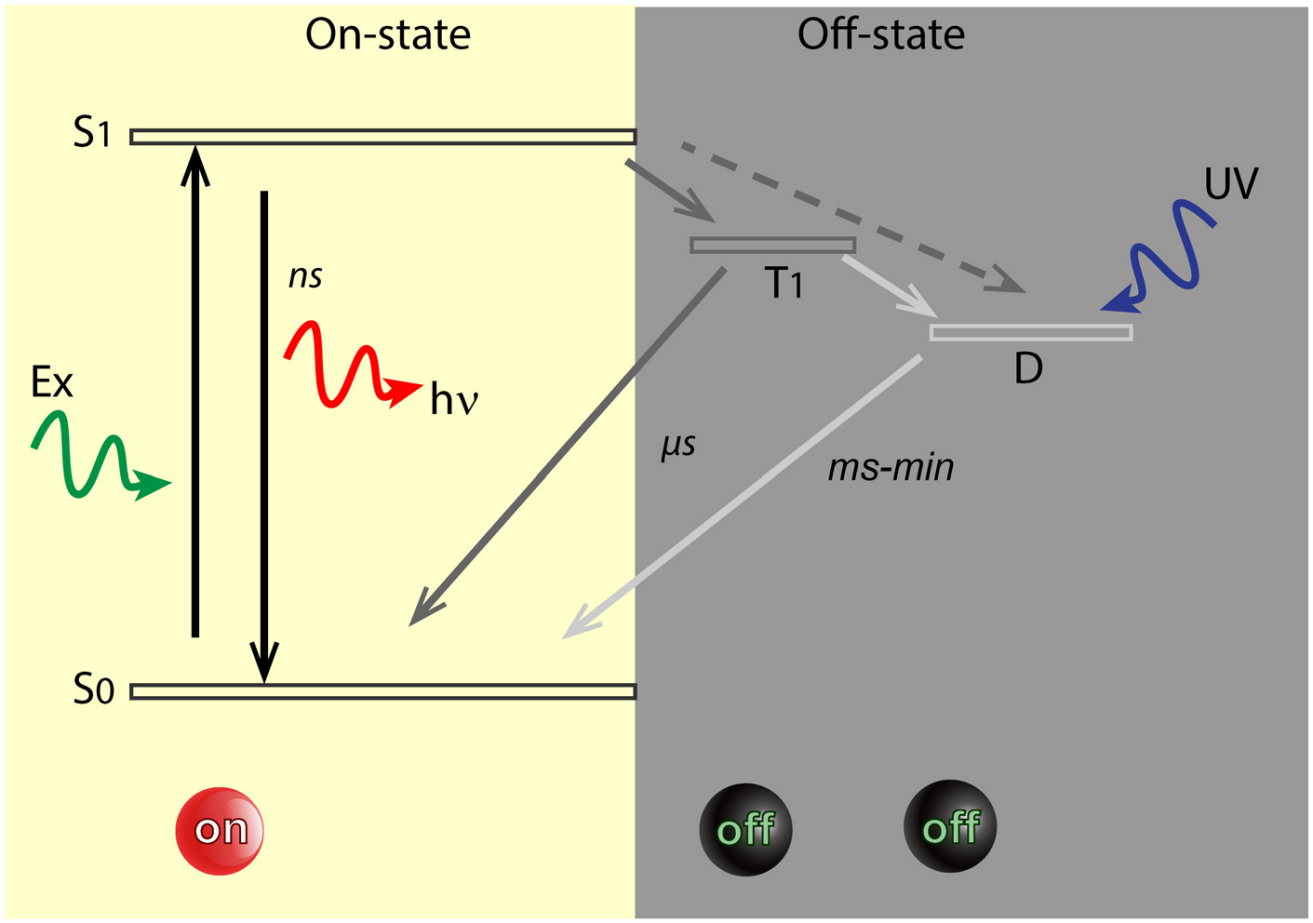
Minimal Jablonski diagram of fluorophore blinking. Simplified Jablonski diagram showing molecular states essential to STORM/GSDIM. For simplicity, neither vibrational levels nor possible additional dark states have been indicated. In the bright On-state, fluorophores can be excited (Ex) from the ground state (S_0_) to the excited singlet state (S_1)_. From there, they may either relax to the ground state by emitting a quantum of light (hν) or alternatively, they may undergo intersystem crossing to the dark triplet state (T_1_). From the triplet state, molecules may return to the ground state or progress to a second, long-lived dark state (D), e.g. through redox reactions. Direct transfer from the excited singlet state to the dark state has also been reported [2] (dashed arrow). Molecules in the dark state may return to the ground state by inverse redox reactions, or alternatively, by exposure to near-UV radiation (back-pumping). S_0_ and S_1_ are called On-states, while T_1_ and D are Off-states. With most molecules in the Off state, it is possible to detect the few remaining bright fluorophores individually in the preparation. Dark states may last between milliseconds and minutes, whereas triplet states may last microseconds at ambient oxygen levels.

Clearly, fluorophore blinking (switching between the on- and off-state) is the vital characteristic that defines a good super resolution (SR) dye. In the on-state, fluorescent dyes are rapidly and repeatedly excited to the excited singlet state (see Fig 1) and, after a few nanoseconds they return to the ground state by emission of red-shifted photons that are detected by the camera. A small fraction of molecules, about 0.1%, may enter a relatively long-lived (μs to ms) non-fluorescent triplet-state. Triplet-state molecules are dark, but eventually return to the ground state and re-enter the normal fluorescence cycle. Not only does the triplet state therefore diminish fluorescence intensity, it also is associated with fluorophore bleaching (the irreversible destruction of the molecule) because in the triplet state a second excitation event renders the molecule highly reactive. Dark states may also be evoked by other mechanisms including rapid and reversible chemical modifications like redox-reactions with oxidants or reductants that are present in the imaging buffer (redox blinking) [1].

Fluorophore blinking has been studied intensely in recent years [1–7] and only a minor fraction of fluorophores tested blink well-enough for efficient SR imaging. A good SR dye:

1. should have high absorption coefficient and quantum yield to produce bright events, because the achievable localization precision scales with the square root of the number of detected photons in the blink event.
2. The *On*-time should be long enough to allow collection of a large number of photons, but not too long because that slows down the data acquisition process.
3. The dye should have a completely dark *Off*-state that is long enough to prevent too many fluorophores to be on at the same time, so as to prevent high background and degradation of S/N.
4. Finally, since in particular 3D imaging requires collection of tens of thousands of images at high laser power, the dye should be exceptionally resistant to bleaching. Bleaching, brightness, on-time and *On/Off* duty cycle are all strongly affected by laser power and buffer composition.

Among the best SR dyes are cyanine derivatives (Cy2, Cy3, Cy5 and in particular Alexa Fluor 647) and rhodamines (Alexa Fluor 488, Atto 488, Alexa Fluor 555 and Alexa Fluor 568) [8]. Unfortunately, these dyes often have widely different requirements for buffer composition. For example, whereas cyanines work optimally in oxygen-free buffers (i.e., they show very little bleaching and display very bright, brief blinks), rhodamines really don’t blink properly in the absence of oxygen [8]. This severely limits our choices for multi-color imaging.

Composition and working mechanisms of imaging buffers have been studied recently [1]. In particular, the concentration of oxygen (O_2_) in the buffers appears somewhat of a double-edged sword. On the one hand, oxygen radicals are responsible for dye bleaching, and therefore O_2_ removal dramatically increases the longevity of the preparation. Therefore, buffers suitable for cyanine dyes typically contain a scavenger system to effectively remove oxygen [1, 3]. On the other hand, however, O_2_ is a major triplet state quencher, that is, the large fraction of fluorophores that ends up in the (dark) triplet state under intense illumination is effectively quenched (brought back to the fluorescent state) by atmospheric O_2_ levels within microseconds. In the absence of O_2_, triplet states may exist for many seconds, strongly reducing the photon flux as well as causing unfavorable blinking behavior [3]. Addition of reducing agents (like MEA, or β-MercaptoEthylamine) at millimolar concentrations may mitigate those disadvantages to some extent by replacing O_2_ as triplet state quenchers, thus restoring high count rate and blinking behavior of the fluorophores. MEA also controls redox-blinking of the dyes, and thus is pivotal in controlling the second (chemical) long-lived dark state (see Fig 1).

To control preparation oxygen levels, several roads have been taken. Embedding in solidifying media such as Poly Vinyl Alcohol (PVA) [9], embedding resin [10], or VectaShield [11] is effective, but it may limit the attainable resolution by e.g. fixing the dipole orientation of fluorophores, causing errors of up to 125nm [12]. Embedding also limits the access of blink-supporting reagents like MEA. For that reason, the highest quality images are still acquired in buffer solutions. The most popular buffer system "Gloxy" uses Glucose-oxidase to consume oxygen in the enzymatic conversion of glucose to gluconic acid. The byproduct of this reaction, hydrogen peroxide, must be removed with catalase because of its adverse effects on fluorophores [13] [8]. In addition, the production of gluconic acid due to ongoing influx of O_2_ in the preparation causes a steady drop in pH (about 2 pH/hours, in our hands) that severely diminishes fluorophore brightness within an hour [14]. This is particularly unfortunate since after mounting, it typically takes up to 20 minutes for the preparation to reach maximal mechanical stability. Acquisition of very large image sets (3D images or large overview-images constituted by stitching of several sub-images) may take up to several hours and is not practical in open dishes. Acidification may be partly remedied by sealing the preparation, for example with the two compound silicone glue Twinsil (Picodent, Wipperfürth, Germany, #13001000) or nail polish [15]. This extends longevity of the preparation to several hours, but eventually the preparations still deteriorate. Long-term storage of such sealed preparations is therefore not feasible because the low pH decreases sample quality in time even without imaging [16].

In view of the above, we concluded that current buffer systems are not optimal for multicolor imaging and they restrict longevity of the preparation. We therefore set out to improve the preparation for prolonged multi-color imaging. In this study, we describe in detail the composition and properties of a new oxygen scavenger system, OxEA, that we recently used for 3-color SR imaging [17]. Here we present a full characterization of OxEA, showing that it does not acidify and that it supports good quality dual- and triple color imaging. We also scrutinized image chamber performance and present a new implementation, based on specially made low-drift glass-bottom chambers from WillCo Wells that can be sealed in an easy and effective way to prevent oxygen influx. With these improvements, we were able to collect good images from a single preparation for several months. We collected an almost unlimited amount of blinking events from the same preparation and even from a single cell, enabling image stitching, extended 3D imaging as well as multi-color recording.

## Results and Discussion

### Part I: OxEA, a new multi-color imaging buffer

In search of a good imaging buffer that support multicolor imaging, we looked in the available literature for oxygen scavenger systems that deplete O_2_ significantly yet do not acidify the medium upon consuming O_2_. Of several different scavengers tested, we obtained best results with Oxyrase, a deoxygenating reagent made of a sterile, oxygen consuming membrane fraction of Escherichia coli and DL-lactate added as a substrate [18]. Note that DL-lactate also has antioxidant and radical scavenger properties itself [19]. To prepare an imaging buffer, the Oxyrase preparation (3% V/V, see Methods) and DL-lactate (100 μM) were dissolved in phosphate-buffered saline (PBS), to which further the triplet state quencher MEA (50 mM) was added. The final buffer, which we termed OxEA (Oxyrase/ β- MercaptoEthylAmine) was adjusted to pH 8.0–8.5 (precise value not important) with NaOH.

**Fig 2:**
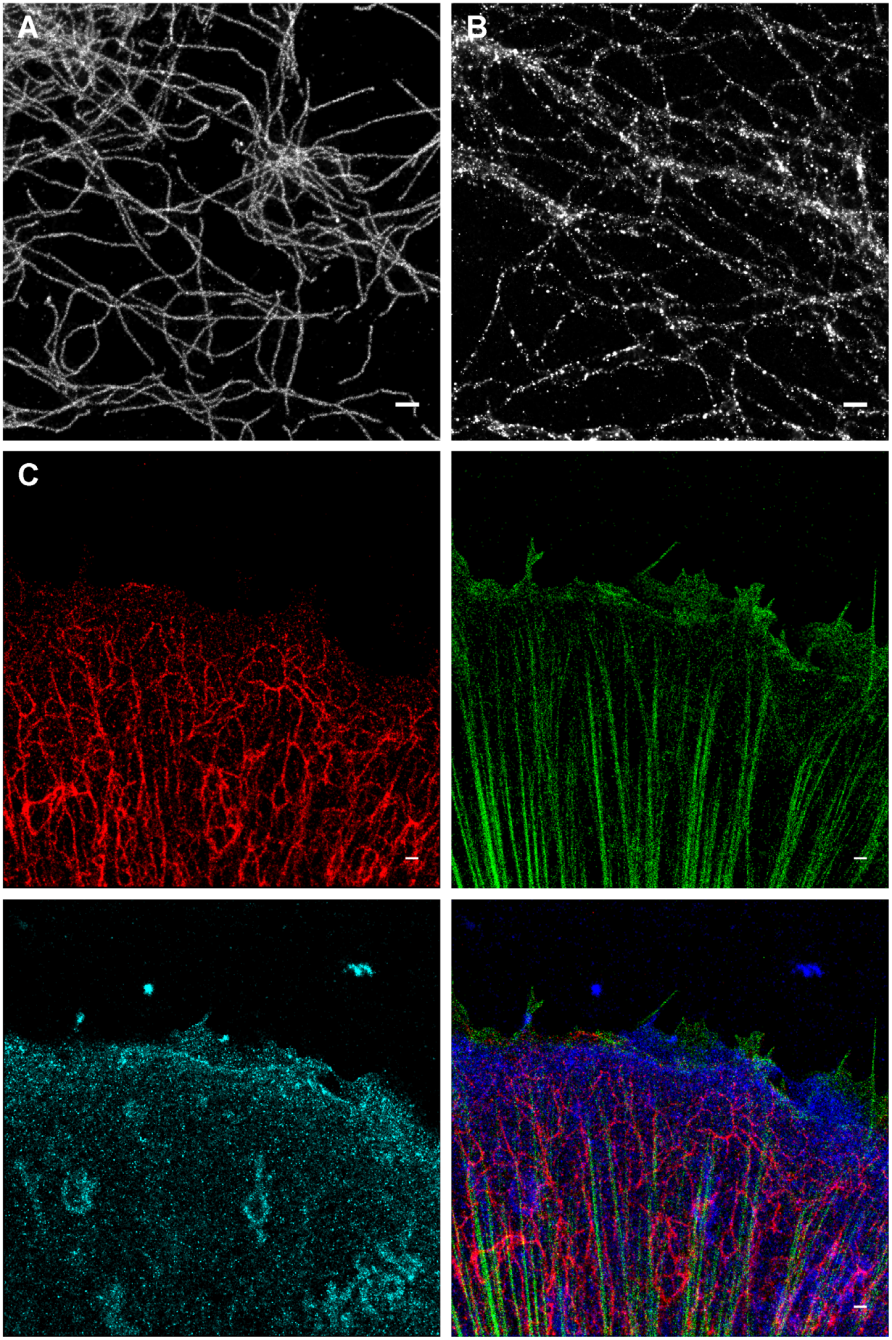
GSDIM imaging in OxEA buffer. (A) comparison of image quality in ageing Gloxy buffer (right) to that in OxEA buffer (left). Images of Ab-labeled vimentin intermediate filaments were collected ~ 2 hours after mounting the preparation in an open dish. (B) 3-color image of keratin (green, Alexa-555), plectin (blue, Alexa-488) and β4 integrin (red, Alexa-647). Approximately 12000 frames where collected for each color channel. Full resolution images are available at https://osf.io/q684r/.

We observed that OxEA supports blinking of Alexa-647 for many hours, yielding very high quality SR images (Fig 2). The image in Fig 2A was acquired 2 hours after mounting of the preparation; for comparison, after two hours in an open dish with Gloxy buffer, many of the fine details in SR images were lost (Fig 2B). Strikingly, unlike Gloxy buffer, OxEA also appeared to support blinking of additional color channels within the same preparation (Fig 2C). We therefore set out to characterize OxEA in further detail.

### Stable pH and oxygen levels in OxEA

**Fig 3:**
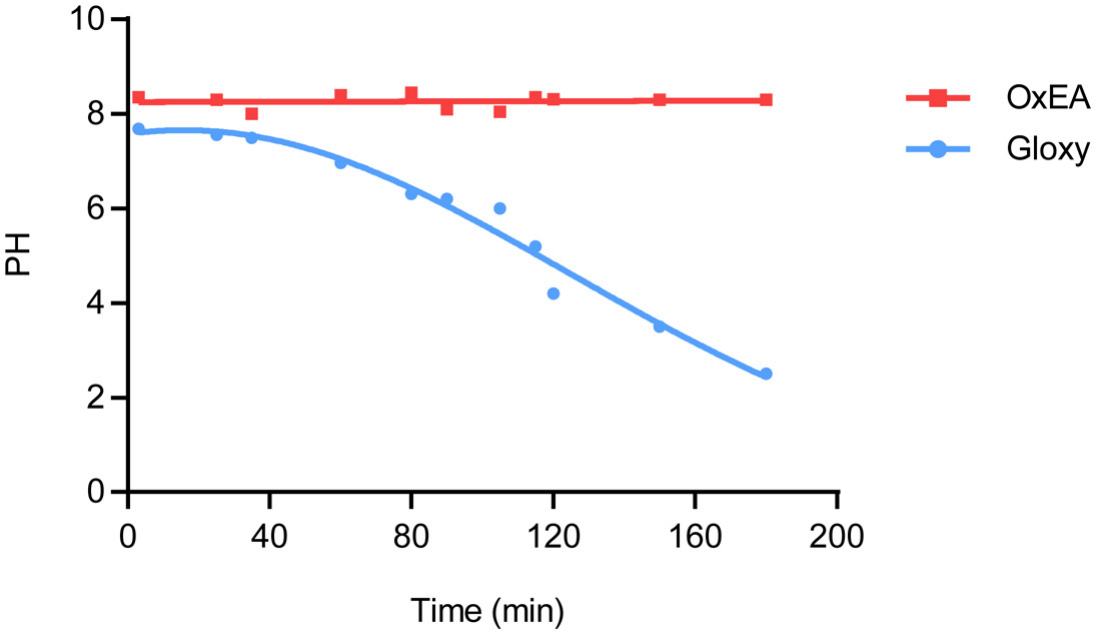
pH in OxEA and Gloxy buffer. The pH in open dishes filled with 0.5 ml of OxEA (red squares) or Gloxy buffer (blue circles) is graphed at the indicated time points. Note the steep drop in pH in Gloxy buffer, which limits imaging to ~ 1 hour unless measures are taken to prevent oxygen influx.

Fig 3 shows that the pH in an open (uncovered) microscopy dish filled with 0.5 ml of Gloxy buffer shows a steady drop at a rate of up to 2 pH units per hour. Since dye brightness and blinking characteristics deteriorate accordingly [14], this effectively limited the time slot for imaging to ~ 1 hour, including the initial 15 min needed to minimize preparation drift. In contrast, the pH detected in OxEA buffer proofed constant, even after several days had passed.

**Fig 4:**
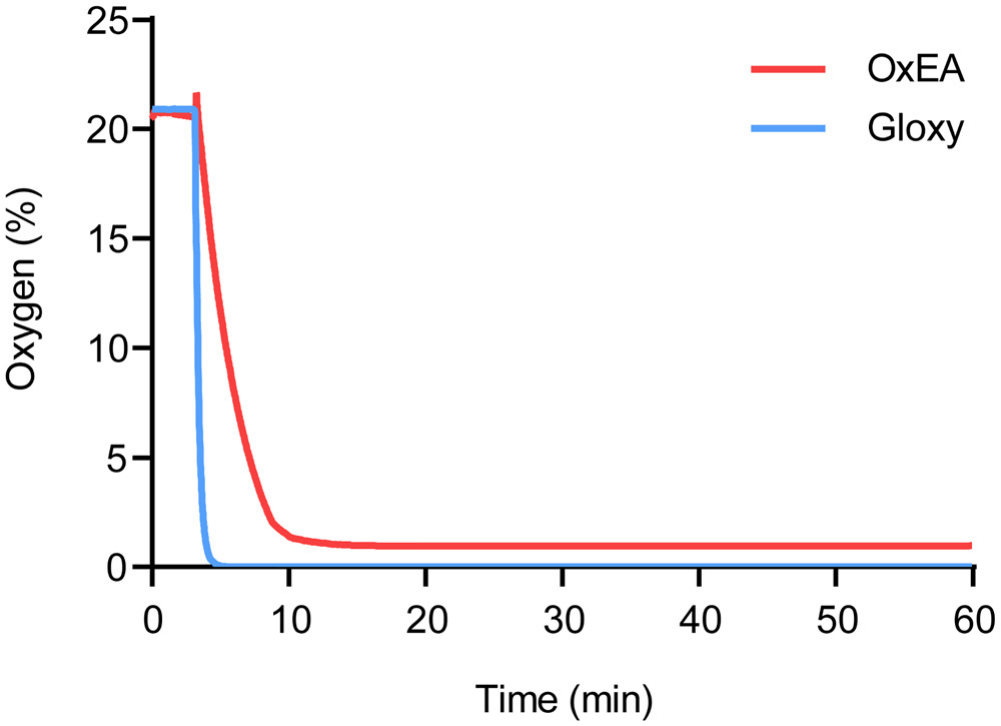
Oxygen levels in OxEA and Gloxy buffers. O_2_ levels were detected every two seconds using a FireSting fluorescence-lifetime based oxygen detector. Note that addition of Gloxy buffer (blue) causes a rapid drop in O_2_ level to undetectable levels, whereas OxEA caused a more slow and less complete removal of O_2_.

Next, we compared the oxygen scavenging properties of OxEA to those of Gloxy buffer. Using a fluorescence lifetime-based O_2_ meter, we recorded oxygen levels in an open dish with PBS continuously at 1 Hz (Fig 4). Addition of Gloxy buffer caused an immediate (i.e., within 20 s) drop in O_2_ levels towards zero, with no detectable free oxygen remaining. Oxygen levels remained below detection limits until the Gloxy buffer ran out, typically after 1–1.5 hours. In contrast, addition of OxEA to the buffer caused a much slower drop in O_2_ levels (Fig 4). Furthermore, OxEA failed to completely deplete O_2_, with some detectable O_2_ remaining (1–2%; Fig 4). We therefore hypothesized that OxEA depletes oxygen sufficiently to cause intense blinking of Alexa-647, while the remaining low (but non-zero) levels of oxygen permit efficient blinking in Rhodamine dyes like Alexa-488 and Alexa-555. Indeed, blinking of Rhodamine dyes (both intensity and number of events above threshold per frame) in OxEA buffer deteriorated markedly when Gloxy buffer was added from a concentrated stock (average intensity 2156 +/- 312 photons/blink in OxEA, versus 371 +/- 99 after adding Gloxy to the dish for Alexa-488). This indicates that incomplete oxygen depletion, rather than one of the other components in OxEA, is supporting the blinking of Alexa-488 and Alexa-555 in these experiments.

### Comparison of blinking and image quality in OxEA and Gloxy buffer

**Fig 5:**
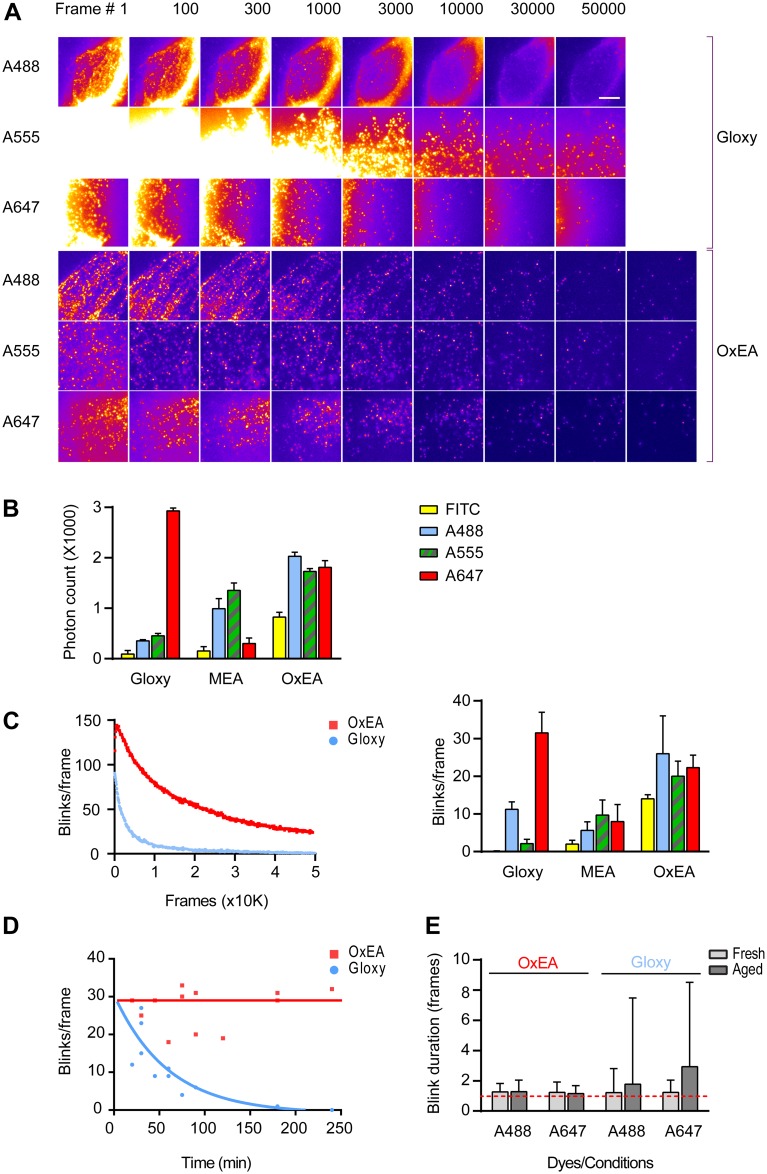
Characterization of blinking in OxEA, Gloxy and MEA buffer. (A) Raw blinking frames (10 ms each, i.e. non-merged results) taken at the indicated time points. Shown are data for Alexa-488 (A488), Alexa-555 (A555) and Alexa-647 (A647) in both fresh Gloxy and OxEA buffer. Imaging was started after a 2–5 second pumping period at full laser power. Note presence of significant structured background in Gloxy buffer. (B) Mean intensity of individual blinks (merged in consecutive frames). Data are mean +/- SEM. See Methods for further details. (C) Left panel, average number of blinks per frame (calculated in blocks of 1000 frames) of a preparation labeled with Alexa-488 and imaged in Gloxy or OxEA. Similar parts of cells with similar initial labeling density were selected based on the low-intensity wide-field image. Note the much larger number of blinks in OxEA for Alexa-488. Right panel, summary of blinks per frame data for Alexa-488, Alexa-555, Alexa-647 and FITC in three different buffers. Data are mean +/- SEM; see Methods for further details. (D) Number of blinks per frame, averaged over the full duration of the acquisition movie, in experiments carried out at the indicated times after applying the buffers. Within the hour, blinking has dropped dramatically in Gloxy whereas OxEA performs well for several hours. (E) Duration of individual blinks of Alexa-488 and Alexa-647 in fresh and ageing (90 minutes) Gloxy, and in fresh and ageing (120 min) OxEA. Multi-frame blinks are very common in ageing Gloxy buffer, as witnessed from the increased average duration of blinks and the enormous increase in duration spread (Data are mean +/- standard deviation). Increased blink duration adds to the appearance of structured background.

For a more quantitative comparison of blinking, we compared cell preparations labeled with different fluorophores in OxEA and Gloxy buffer. RF EC24 endothelial cells were labeled for vimentin using a mouse monoclonal antibody (Ab) and secondary Ab labeled with Alexa dyes. Series of 50,000 frames or more were collected in TIRF mode at 100 fps, and we calculated blink intensity, blink duration and the number of blinks per frame as a function of imaging time. Fig 5 shows representative data as well as summary statistics. Our data can be summarized as follows.

First, whereas in Gloxy buffer excitation with the 647 laser depleted the ground state of Alexa-647 in about 10 s and caused very bright blinking for prolonged times (Fig 5A, Gloxy buffer, lower strip), in this buffer other dyes including Alexa-488 proved much harder to pump to the dark state and blinks were much less bright and defined. The latter is likely due to a conspicuous background that may be attributed to poor ground state depletion (Fig 5A). A similar observation was made for Alexa-555 (Fig 5A). In OxEA buffer, Alexa-647 performed fine in that it was quickly pumped to the dark state (Fig 5A) and blinked well for a prolonged time, although the blink intensity was somewhat diminished (Fig 5B) as compared to Gloxy. In contrast to Gloxy buffer, excitation in OxEA readily depleted the ground state of Alexa-555and Alexa-488 (Fig 5A), resulting in images with considerably less background. Blink intensity of Alexa-488 and Alexa-555 also proved higher in OxEA buffer (Fig 5B) resulting in superior images (Fig 2).

Second, in Gloxy buffer the number of events per frame typically drops to unpractically low levels after about 10 to 20 thousand frames (Fig 5C), whereas in OxEA typically a higher blink count is observed at least up to 50,000 frames. This is especially important for collection of 3D images because these require much more frames for a complete reconstruction. In STORM/GSDIM, back-pumping with a 405 nm laser has been commonly used to partially compensate for ongoing run-down of blink rates. In none of the experiments included in this report back-pumping was applied; however, it is important to note that 405-nm back-pumping worked approximately equally effective on blinking rates in OxEA and Gloxy buffers.

Third, whereas Gloxy buffer is best used fresh and deteriorates noticeably already after 30–45 minutes (Fig 5D; note that this is particularly apparent in the Alexa-488 and Alexa-555 channels), OxEA supports good blinking for at least four hours in open dishes. This makes it much more convenient to record several images from the same sample. We noted that in ageing Gloxy buffer, individual events became much less bright. Even more importantly, fluorophore on-time changed significantly, showing both an increased mean on-time as well as a dramatic increase in variability due to emergence of a population of blinks that spanned many (> 10) frames (Fig 5E).

Fourth, it is noteworthy that OxEA also supports good blinking of labels that have not been used very successfully in GSDIM/STORM imaging, such as FITC (Fig 5B). This is important because it enables selecting from the large collection of (primary) antibodies that have been labeled with this popular dye over the years.

We also assessed other properties that could potentially deteriorate final image quality in OxEA, when compared to (fresh) Gloxy. Apart from a slight decrease in localization precision for Alexa-647 (due to lower photon count; compare Fig 5B), we identified no factors that could lead to diminished image quality, and the FWHM profiles of the smallest discernible keratin filaments were comparable for Alexa-647 (41 +/- 7 and 42 +/- 9 nm, mean +/- standard deviation of 10 measurements for OxEA and Gloxy each, respectively).

In summary, the combination of fresh Gloxy buffer and Alexa-647 still provides the highest possible localization precision, but OxEA appears to be a superior buffer for low-background multi-color recording and when preparation longevity is important. The 3-color image presented in Fig 2 demonstrates the fine quality of 3-color images that can be routinely obtained with OxEA buffer.

### Part II: Keeping oxygen out: a simple and stable oxygen-tight chamber

In the above, open dishes exposed to ambient air have been compared for the two buffer systems. Several reports have shown that the longevity of Gloxy buffer preparations can be significantly increased by covering and sealing the preparation, which is particularly important with cyanine dyes. Preventing oxygen influx abolishes acidification and peroxide production and therefore averts the main shortcomings of Gloxy buffers, at least for single-color imaging. In testing these solutions we noted that indeed, sealing the preparation with two-component compounds like Twinsil can increase preparation lifespan to 4–24 hours although eventually, the quality of the images still deteriorates. Furthermore, sealing often significantly increased preparation drift. This is probably due to differences in expansion coefficient between the aqueous buffer and the glass coverslips, which causes small temperature differences to generate stress on the coverslip. Indeed, a night in the refrigerator is often enough to draw small air bubbles in sealed preparations. We therefore next addressed drift and sealing of the recording chambers.

### WillCo Well # GWSB-3512-N: a dedicated low-drift SR glass bottom dish

**Fig 6:**
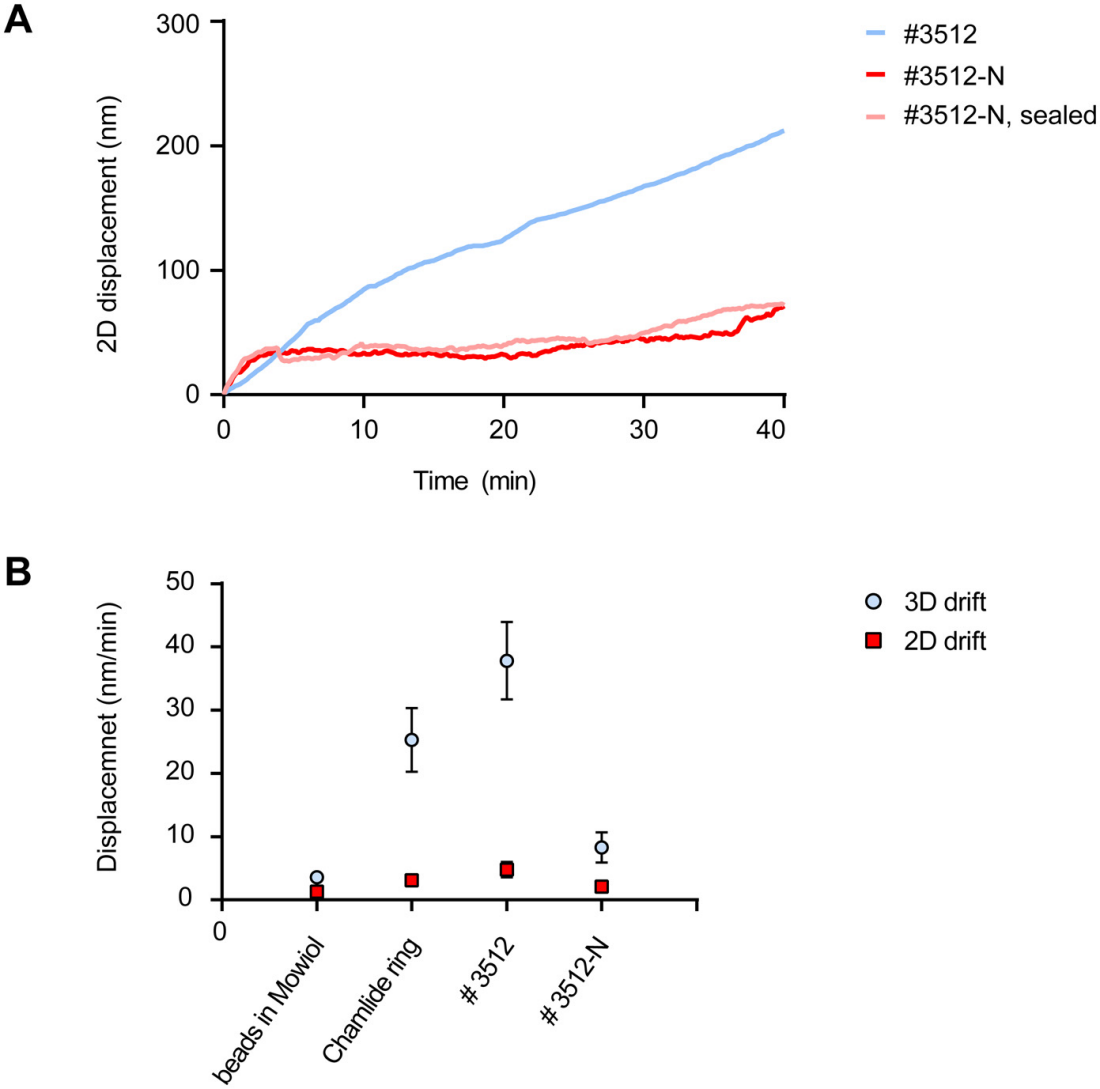
Optimizing drift in SR preparations. (A) Example traces of drift quantifications during 30 min in #3512 dishes (blue), #3512-N dishes (red) and #3512-N dishes sealed with coverslip and adhesive-backed aluminum tape (pink). Shown is the mean displacement away from the origin of immobilized beads during 30 min. (B) Summary of 2D (lateral; square symbols) and 3D (lateral + focus, round symbols) drift experiments. Shown are endpoint drifts at 30 min and at 60 min for the indicated imaging dishes. The WillCo Wells optimized #3512-N dishes display significantly improved stability. Data are mean +/- SEM of >3 experiments each.

Drift was characterized in detail using glass surfaces with sparse sub-resolution fluorescent beads immobilized to them, by imaging for extended time at low power excitation on our Leica SR-GSD microscope. The microscope is located in a separate air-conditioned room with temperature stability of +/- 1 degree C. X, Y and Z position were detected to nm precision in 3D-acquisition mode (see Methods). The drift of the microscope SuMo stage itself appeared 1.3 +/- 0.7 nm/min in X/Y and less than 1.6 +/- 1.1 nm/min in 3D (X,Y and Z combined) after allowing an initial settling time of 15 min (Fig 6B). Maximum excursion from the starting point observed over a 2-hour period was 35 nm in 3 dimensions. In contrast, over a period of 30 min, various glass bottom dishes from commercial sources showed drift of up to 700 nm in X/Y and 1.2 μm in Z, often rendering the data of 3-D stacks completely useless. We tested single-well chambers as well as 8-well slides. Although not systematically quantified, sealing with Twinsil appeared to further deteriorate preparation stability.

Glass-to-plastic bonding may be suspected to be a prime source of drift due to differences in thermal expansion coefficients, but also because curing of the bonding material generates visible stress (bending) of the coverslip. In response to our observations WillCo Wells, a leading producer of microscopy glass-bottom dishes for in-vitro fertilization purposes, developed alternative curing protocols for the adhesive which resulted in significantly diminished bending of the glass coverslips. We found that type ***GWSB-3512-N*** WillCo Wells perform extremely well in drift tests (Fig 6), drifting on average only 2.1 nm/min in XY and 8 nm/min in X, Y and Z. This represents an improvement of at least 4-fold (and often much more) over other commercial dishes tested in our lab. We conclude that glass-to-dish bonding is extremely important to avoid preparation drift and suggest WillCo Wells ***GWSB-3512-N*** as a good commercial source of low-drift dishes.

Finally, we also tested several cover slip mounting rings for drift. Best results were obtained with reusable Live Cell Instruments magnetic coverslip mounts (Fig 6B). Much more drift was observed with screw-on type rings, probably because they do put more strain on the cover slip. Importantly, any slight residual drift observed in the optimized preparations could be easily corrected for in software, either through inclusion of fiducial markers or through the piece-wise image correlation algorithm present in ThunderSTORM software.

### Improved sealing blocks oxygen influx: near-unlimited blinking

**Fig 7:**
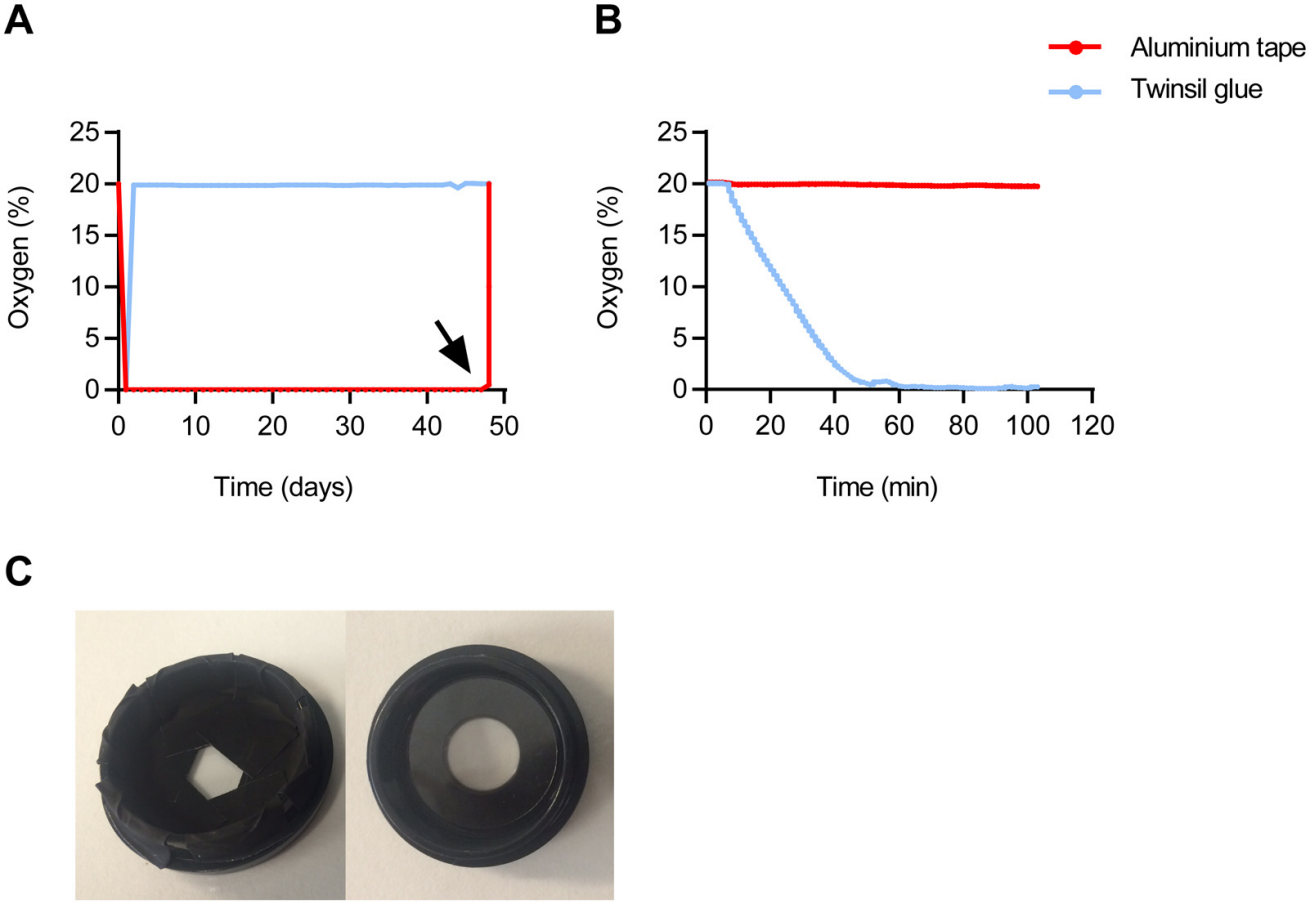
Sealing cell culture dishes to prevent oxygen influx. (A) O_2_ levels, detected by daily fluorescence lifetime-based recording in a WillCo Well sealed with Twinsil glue (blue) or aluminum tape (red), respectively. At 48 days, the seal was broken to test responsiveness of the O_2_ sensor (black arrow). (B) A fluorescent O_2_ indicator pad was covered with Twinsil (blue) or aluminum tape (red) and submerged in buffer at ambient oxygen levels. O_2_ levels were recorded continuously, and at t = 8 min oxygen scavenger was added from concentrated stock. Note the rapid drop in O_2_ levels below the Twinsil seal, indicating its permeability to oxygen. (C) WillCo Wells #GWSB 3512-N dish shown with 24-mm coverslip lid (right) and sealed with pieces of aluminum tape (left).

Several protocols have been reported to prevent oxygen influx by covering and sealing with adhesives or vacuum grease. Significant improvements were obtained when the cover glass was sealed using e.g. vacuum grease, parafilm [20] or with Twinsil, a two-component elastic adhesive [15]. This increased longevity of the preparations several fold, but eventually (within hours or days) preparations still run down. Strikingly, when tested with the fluorescence lifetime O_2_ logger, we observed that Twinsil is quite permeable to O_2_ (Fig 7A and 7B). We therefore tested a range of fast-curing adhesives including UV-curable glass glue, nail varnish, and various varieties of epoxy glue and silicone kit. None of these were completely effective in blocking O_2_ influx. In addition, like with Twinsil, application of these sealants frequently caused additional drift.

In industrial process technology, adhesive-backed aluminum metal foil is successfully used to seal sutures and prevent gas exchange. In O_2_-lifetime experiments, this material proved fully impermeable to oxygen (Fig 7B). Indeed, it proved easy to completely seal WillCo Wells as well as other containers (Fig 7C) and such preparations retained very low O_2_ levels for periods of up to several months.

**Fig 8:**
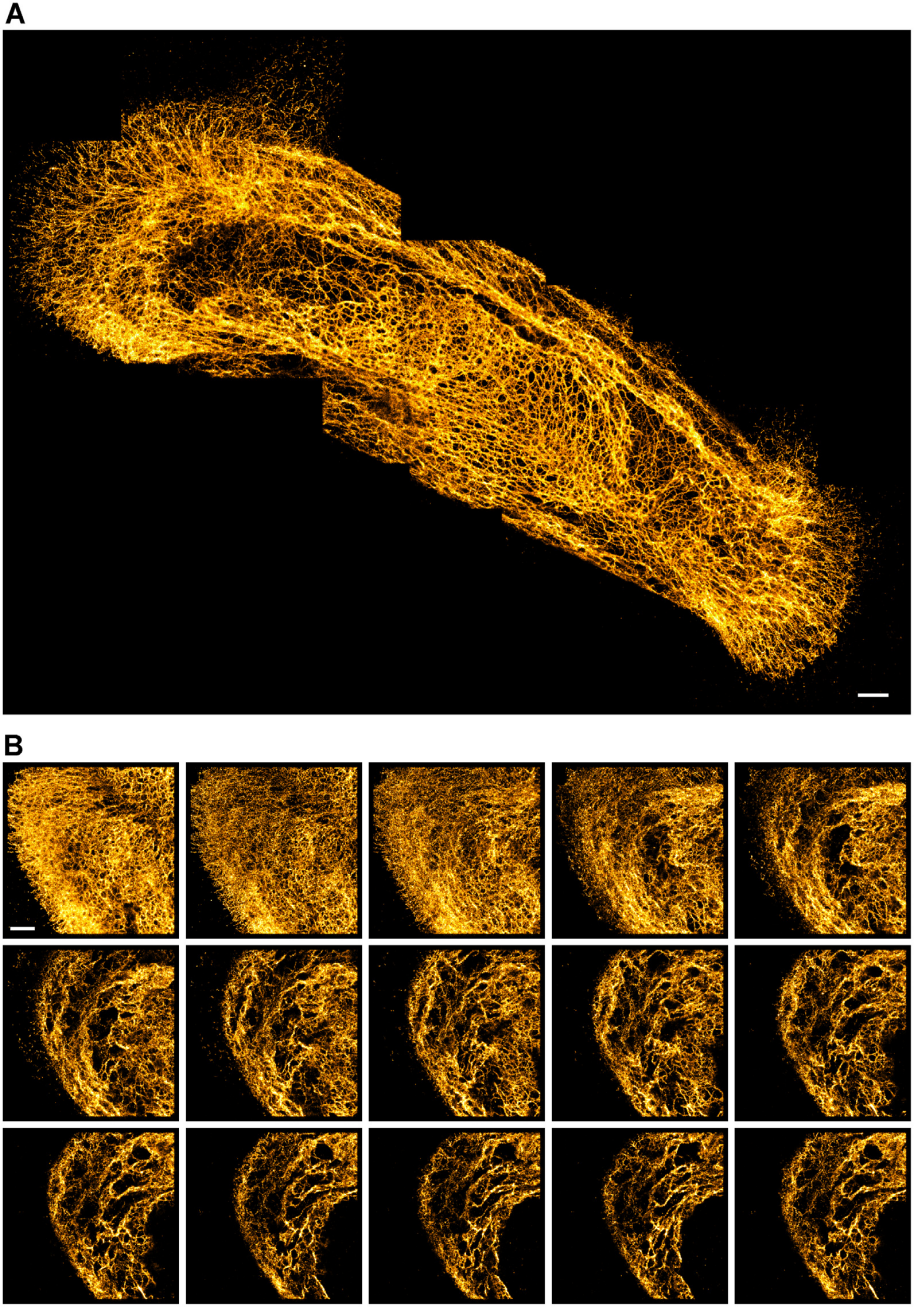
OTC enables near-unlimited blinking of Alexa-647. (A) Stitched image, composited of eight 1800x1800 pixel images (each based on 20k raw blinking images) showing the keratin cytoskeleton in a HUVEC cell. Despite partly overlapping acquisition areas, almost no loss in image quality was noticeable throughout the experiment. (B) Z-stack of keratin cytoskeleton in PA-JEB/β4 keratinocytes. Consecutive images were taken after refocusing the lens by 300 nm manually. The full-resolution images will be available at the associated data repository (https://osf.io/q684r/).

This oxygen-tight chamber (OTC) preparation allows recording of exceptionally long series of frames from a single cell. For example, the image in Fig 8A is stitched from 8 individual SR images, each collected with >20,000 frames. In S1 Movie we provide a zoom-in on the last image that was collected in this series, showing that even after extensive exposure of this area, the last image still contains excellent detail. In Fig 8B we present a 3D stack consisting of 15 images that required a total or 300,000 frames to be collected from the same area of the cell. The total collection time in this experiment was ~ 1.5 hours. To push repeated imaging to the limit, we tested collection of 1,5000,000 frames in a single 180x180 pixel image. This still did not terminally bleach all fluorophores. Finally, we note that the OTC also makes it feasible to revisit imaged cells days or weeks later, which is useful when additional data are needed on e.g. the cellular context of a previously recorded image. In practice, preparation longevity was limited by dissociation of the antibodies/ labels from the epitopes. In conclusion, oxygen tight chambers present convenient preparations in all cases where preparation longevity is vital.

## Conclusion

We have addressed several critical steps in preparation and image acquisition of localization microscopy. First, we formulated and tested a new imaging buffer, OxEA, which routinely supports blinking of several dyes for multi-color imaging. OxEA works well with a number of dyes commonly used in SR, including Alexa-488, Alexa-555, Alexa-647 and Cy5. Moreover, it supports blinking of FITC, which is advantageous because many laboratories have stockpiles of FITC-labeled antibodies.

A good buffer for multi-color SR imaging should support effective pumping of dyes to the dark state, bright blinking, a good ratio between on- and off-state durations, and it should prevent terminal bleaching of the dyes so as to allow acquisition of very large series of frames. In addition, the buffer should not run down or acidify. In Gloxy buffer, protection against acidification has been improved by inclusion of 50 mM TRIS buffer (pH 8.0) [14]; however, Alexa-532, a second labeling color often used in recent literature, does not blink well under these circumstances (see S1 Fig). While it is clear that OxEA does not acidify or run out as fast as Gloxy buffer, it is difficult to point out one single property that explains why OxEA supports multicolor blinking so well. Our results indicate that the pumping phase proceeds fast and efficiently in OxEA. This results in less (structured) background in the raw images, which increases the number of above-threshold blinks. Compared to Gloxy buffer, blinking intensity is also augmented for Alexa-488 and Alexa-555, although we note that the combination of fresh Gloxy buffer with Alexa-647 still yields highest intensity blinks. Furthermore, OxEA also protects dyes against terminal bleaching, as we were routinely able to collect >100.000 frames from the same cell. This result indicates that individual fluorophores also re-blink more frequently in OxEA buffer, although this aspect is difficult to quantify and has not been addressed systematically in this study. In ageing Gloxy buffer, blinking properties of Alexa-488 appear to change considerably (Fig 5E), i.e., the majority of blinks span many frames. We also observed blink intensity to drop considerably, although this appeared somewhat variable. In contrast, during extended time series in OxEA, blinking properties were much more stable.

While testing various methods to seal preparations to prevent oxygen influx we observed that the commonly recommended two-component sealant Twinsil is rather permeable to oxygen. Instead, we showed that adhesive-backed aluminum foil performs well for this application. In such oxygen-tight preparations, we were able to record near-unlimited numbers of frames, which allowed us to constructs very high density 3D stacks and large stitched images.

Finally, we addressed preparation drift in some detail. These studies identified two low-drift solutions. First, for mounting 24 mm coverslips we found that a magnetic coverslip mount, Live Cell Instruments Chamlide (CM-B25-1) performs significantly better than screw-type mounting rings, probably because the latter cause strain in the coverslip. In addition, together with WillCo Wells, a disposable solution has been developed and tested. In these dishes, improved bonding of the coverslip to the plastic dish remedied most of the drift. Both of these preparations can be sealed effectively with adhesive-backed aluminum tape to construct oxygen-tight chambers for prolonged imaging.

In summary, we have introduced incremental improvements in imaging buffer, measurement chamber and sealing methods to improve both multicolor SR imaging and preparation longevity significantly.

## Materials and Methods

### Cell culture

Ultra-clean coverslips were prepared by acid/base rinsing as follows: first, nr 1.5 coverslips (Thermo Fisher Scientific, Waltham, USA) were washed in 2M HCl overnight, followed by a 2-hour wash in 2M NaOh and an additional wash in 20% H_3_PO_4_. Inbetween those steps, coverslips were briefly rinsed with double-distilled H_2_O. Storage until further use was in ethanol. For imaging, immortalized Human Vascular Endothelial Cells EC-RF24 [21] or PA-JEB/β4 keratinocytes [22] (were seeded on either ultra clean coverslips or Wilco well glass bottom dishes (Amsterdam, The Netherlands) optimized for SR (***GWSB-3512-N***). The latter dishes we found to be clean enough for SR imaging without the need for acid/base rinsing. EC-RF24 cells were grown in Medium 200 (M-200-500, Thermo Fisher Scientific) with the addition of Low Serum Growth Supplement (LSGS, Thermo Fisher Scientific, Waltham, USA) until they reached 50% confluency and then fixed with 10% MeS buffer (100 mM MeS, pH 6.9, 1 mM EGTA and 1 mM MgCl_2_) and 90% methanol for 5 min on ice. PA-JEB/β4 cells were fixed with glutaraldehyde to preserve actin structure (incubation with 0.3% glutaraldehyde + 0.25% Triton in a buffer containing 10 mM MES pH 6.1, 150 mM NaCl, 5 mM EGTA and 5 mM MgCl_2_) for 2 min, followed by 10 min fixation in 0.5% glutaraldehyde in the same buffer (no Triton present). Subsequently, the sample was treated with freshly prepared 0.1% NaBH_4_ for 10 min. After blocking with 5% Bovine Serum Albumin (BSA; Serva, Heidelberg, Germany) for 1 hour, cells were stained as follows. For EC-RF24 cells were incubated with mouse anti-vimentin monoclonal antibodies (code No. M 0725 Clone V9, Dako, Heverlee, Belgium). Subsequently all the cells were incubated with goat anti-mouse antibodies (Alexa-488, Alexa-647, Alexa-555, Alexa-532 or FITC, Thermo Fisher Scientific) at a final concentration of 0.01 mg/ml for 30 minutes. For PA-JEB/β4 cells staining was with rabbit anti-keratin 14 polyclonal antibody (Covance, Princeton, USA), rat anti-β4 (BD biosciences, Breda, The Netherlands) and Phalloidin conjugated to Alexa Fluor 647 fluorophores (Invitrogen). Samples were incubated with goat anti-rabbit and goat anti-rat secondary antibodies labeled with Alexa-488 and Alexa-555 fluorophores (Invitrogen) afterwards. All the fixation and staining steps were done at room temperature.

The Coverslips were mounted in a holder (Chamlide CMB mounting ring CM-B25-1, Live Cell Instrument, Seoul, South Korea) filled with 500 μl of imaging buffer (see below). After mounting the preparation on the microscope, we waited for ~15 min in order for the preparation to stabilize before starting imaging. In experiments aimed at assessing buffer quality, this waiting time was skipped and any drift was compensated for in software.

For imaging in O_2_-tight sealed Willco Wells (OTC), first the glass bottom was covered with ~ 100 μl of Gloxy buffer. A rinsed coverslip was placed as a lid on the top of the well using a tweezer, taking care to avoid inclusion of air bubbles in the imaging medium. Then the excess buffer was dried carefully, and pieces of black non-reflective aluminum tape T205-1.0—AT205 (THORLABs Inc, Newton, New Jersey, USA) were applied in a partly overlapping manner and carefully pressed down to seal the preparation.

### Imaging

Samples were imaged on a Leica SR-GSD microscope (Leica Microsystems, Wetzlar, Germany) equipped with 488 nm/300 mW, 532 nm/500 mW and 642 nm/500 mW lasers and an EMCCD camera (Ixon DU-897, Andor). We used a 160x oil immersion dedicated SR objective. For three-dimensional images the Leica astigmatic lens was used.

Between 10.000 to 100.000 frames were collected at 100 Hz with image size of 180×180 or 400×400 pixels. The data sets were analyzed with the Image J ThunderSTORM analysis module [23] and images were reconstructed with a detection threshold of 200 photons, options sub pixel localization of molecules and uncertainty correction checked, with a rendering pixel size of 10 nm. Software drift correction (ThunderSTORM) was applied, using either the image cross-correlation algorithm (Nr of bins = 10; magnification = 5), or alternatively, by using fiducial markers, for which sub-resolution fluorescent beads were included in the preparation.

For multi-color images, first Alexa-647 was imaged, followed by Alexa-488 (using a 500/30 band-pass filter to prevent leak-through of Alexa-555). After Alexa-488 was largely bleached due to long-term imaging the third channel (Alexa-555 or Alexa-532) was imaged.

Series of raw blinking images were first subjected to running-median background subtraction [24]using an in-house developed Image J macro. All images where further corrected for chromatic aberrations.

### Composition of the imaging buffers

1. OxEA
  - 50 mM β-MercaptoEthylamine hydrochloride (MEA, Sigma-Aldrich)
  - 3% (v/v) OxyFlour^™^ (Oxyrase Inc., Mansfield, Ohio, U.S.A.)
  - 20% (v/v) of sodium DL-lactate solution (L1375, Sigma-Aldrich)
  - in PBS, pH adjusted to 8–8.5 with NaOH
2. Gloxy buffer
  - 50 mM β-MercaptoEthylamine hydrochloride (MEA, Sigma-Aldrich)
  - 10% (v/v) of a 250 g/l solution of glucose
  - 0.5 mg/ml glucose oxidase
  - 40 mg/ml catalase (Sigma-Aldrich)
  - in PBS, pH 7.6
3. MEA buffer
  - 50 mM β-MercaptoEthylamine hydrochloride (MEA, Sigma-Aldrich)
  - in PBS

All buffers were prepared freshly in 500 μl aliquots before imaging unless otherwise noted in the text. According to the supplier, repeated freezing/thawing is detrimental to Oxyrase solutions. For pH buffering, we prefer phosphate buffers over HEPES buffers (because of the unfavorable redox behavior of HEPES) or TRIS buffers (unfavorable pKa because it should give maximal protection against acidification in Gloxy).

### Analysis of blinking characteristics

Assessment of dye blinking properties was carried out as follows. Series of raw images were background-corrected and analyzed using ThunderSTORM with default settings, except: magnification = 10x to obtain final pixel size of 10 nm. Result files (*.csv) where then subjected to drift correction (using either a self-written Image J macro or the ThunderSTORM build-in drift correction option) and blinks present in consecutive frames were merged in 2D (maximum distance = 20 nm, maximum off-frames = 1). For analysis of blinking brightness (Fig 5B) 100,000 blinks were averaged per movie in SPSS statistical software. Data are average +/- SEM from 3–15 individual experiments for each condition, using different batches of buffers and on at least 2 days of experimentation. Analysis of the number of blinks per frame (Fig 5C and 5D) was based on comma-separated value (.csv) files corrected as indicated above, using a home-written analysis routine in Visual Basic.net to calculate total number of blinks in blocks of 1000 raw frames. Visualization of blink duration (Fig 5E) was by manually drawing small ROI around blinks in a given frame, followed by plotting intensity in the ROI in frames -50 to +50 with respect to the given frame using Image J <Stack / Plot Z-axis Profile> option.

We note that it is not trivial to determine these blinking properties precisely because the relevant properties are not necessarily independent. For example, the presence of significant structured background in Gloxy buffer may result in an underestimation of the number of detected blinks per frame in the first part of the time-series. For that reason, we have refrained from statistical testing of the observed differences.

### Quantification of preparation drift

For quantification of drift, we first quantitated stability of the Leica GSDIM microscope with SuMo Stage using 0.4 μm fluorescent TetraSpeck microspheres (Invitrogen, Waltham, Mas., USA) immobilized on thick preparation slides and embedded in Mowiol. For drift measurements in imaging chambers, beads were immobilized to the glass coverslip by air-drying a diluted solution. In all cases, mean displacement away from the origin was determined using an Image J macro to find the center of intensity in each of the beads and then averaged over all beads in the image. After an initial stabilization period of 15 min, images were collected every second for the duration of one hour at low intensity excitation. Drift was expressed in nm/min and total (accumulated) drift was also calculated for a period of 30 min and for 60 min. During these experiments the room was kept closed because we observed that draft may significantly degrade stability.

### Detection of dissolved Oxygen levels

Dissolved oxygen levels were measured using a calibrated FireSting O_2_ meter (PyroScience, Aachen, Germany) according to the manufacturers guidelines. We used OXSP5 sensor pads which work both in dry and submersed condition. In the experiments for Fig 7B, sensor pads were stuck to the dish, then wetted and covered with either Twinsil of adhesive-backed aluminum tape. Dishes with sealed sensors were then filled with water. Continuous O_2_ measurements were started from the submerged sensor pads and after about 10 minutes, oxygen scavenger (either Gloxy or 50 mM of NaSO_3_) was added.

## Supporting Information

S1 Fig Alexa-532 brightness increases with acidification. Shown are the average photon count per blink of Alexa-532 (red) and the buffer pH recorded during 3 hours of ageing in Gloxy buffer. Note that the ongoing drop in pH increases the brightness of Alexa-532 by almost two-fold.
(TIF)

S1 Movie Zoom-in on a stitched image covering the keratin cytoskeleton of a HUVEC cell. The image is constructed from eight individual SR images consisting of >20,000 frames each. Images were acquired consecutively; zoom-in is on the last acquired image.
(AVI)

## Abbreviations

SMLM: Single Molecule Localization super-resolution Microscopy
STORM: Stochastic Optical Reconstruction Microscopy
GSDIM: Ground state depletion followed by individual molecule return
PALM: Photoactivated localization microscopy
SR: Super resolution
EM-CCD: Electron multiplying charge coupled device
CMOS: complementary metal-oxide semiconductor
A488: Alexa Fluor 488
A555: Alexa Fluor 555
A532: Alexa Fluor 532
A647: Alexa Fluor 647
MEA: β-MercaptoEthylamine
OTC: Oxygen tight chamber
S/N: Signal to Noise ratio
PVA: Poly Vinyl Alcohol
OxEA: Oxyrase/β-MercaptoEthylamine
SEM: Standard Error of the Mean
SuMo stage: Suppressed Motion stage
