# Supplementary figures and images for: Optimizing Imaging Conditions for Demanding Multi-Color Super Resolution Localization Microscopy
Source: PLoS One. 2016 Jul 8;11(7):e0158884. doi: 10.1371/journal.pone.0158884 (PMC4938622; doi:10.1371/journal.pone.0158884)

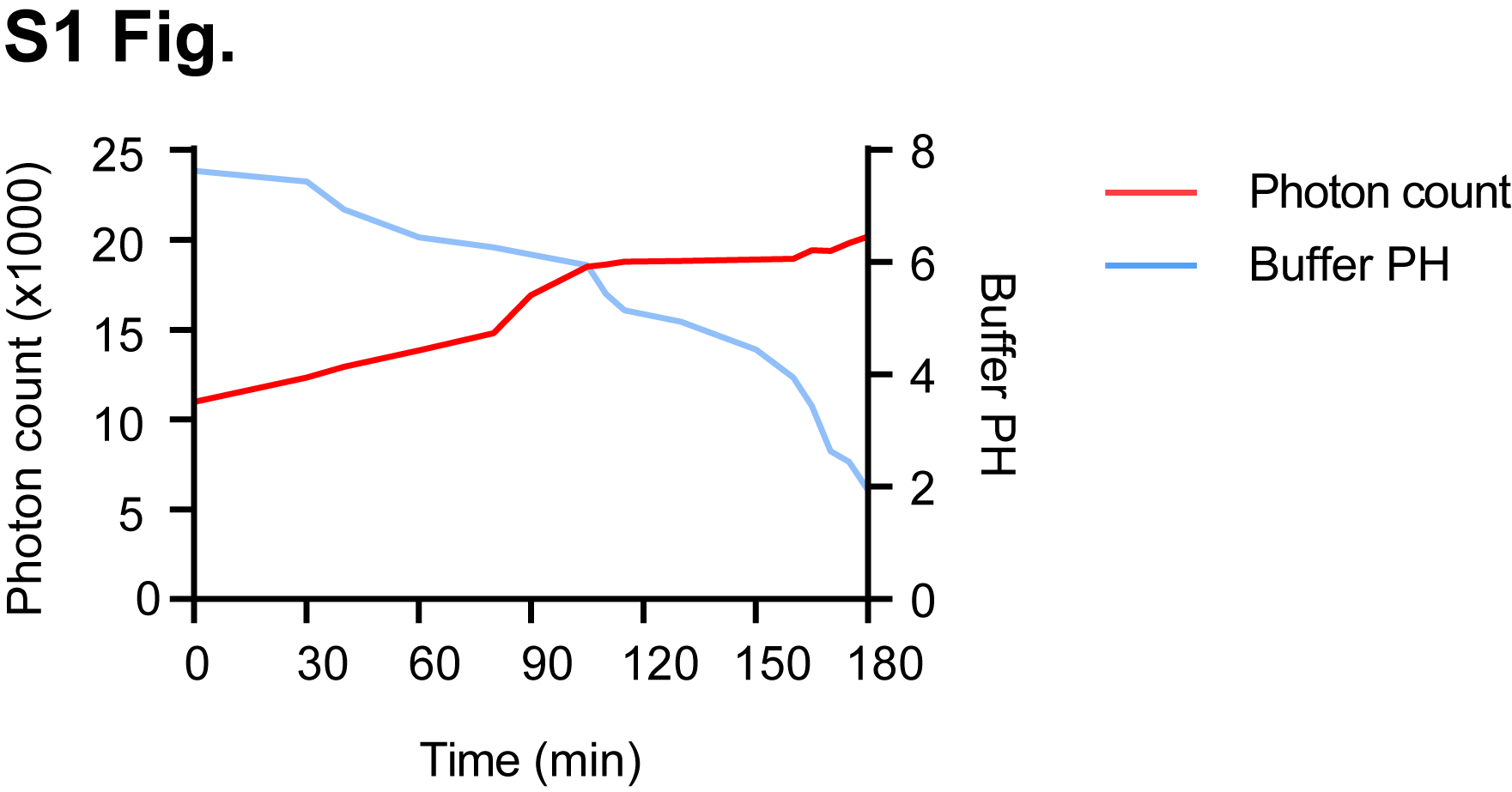

Supplement: S1 Fig — Shown are the average photon count per blink of Alexa-532 (red) and the buffer pH recorded during 3 hours of ageing in Gloxy buffer. Note that the ongoing drop in pH increases the brightness of Alexa-532 by almost two-fold. (TIF) [file pone.0158884.s001.tif]
